# Abnormal Fœtus

**Published:** 1865-07

**Authors:** F. R. Payne

**Affiliations:** Marshall, Illinois


ARTICLE XXVI.

### ABNORMAL FŒTUS.

By F. R. PAYNE, M.D., Marshall, Illinois.

We recently met with a very remarkable case of an abnormal fœtus, a brief history of which we propose to present to the readers of your Journal.

These freaks of nature are not uncommon; but this case presents a very important fact for the consideration of those engaged in physiological investigations.

It is here demonstrated that a child can live and grow in utero almost to its normal size without a single trace of brain or spinal marrow.

On the 10th day of April, 1865, I was called in haste to see Mrs. D., the mother of four children. She was as supposed eight months advanced in pregnancy, and unnaturally abdominally enlarged. The liquor amnii was thrown off in large quantity, supposed to be at least two gallons, but no uterine pains were felt at the time of the rupture of the membrane. She was not alarmed, but sent for me, with the expectation of the rapid approach of labor. For the last eight weeks, she has suffered greatly; and could not sleep only in her chair or propped up in bed. She had, during this time, excruciating pain in her left side and hips; her feet were unusually edematous. After the discharge of liquor amnii, she could rest comfortably in bed in the recumbent posture; and the pain of her side and hips was relieved.

On the 12th day of April, she felt slight uterine pains, and before assistance reached her a monstrosity was born; also a natural and healthy placenta. She had felt the motion of the child up to the day before it was born. After the birth of the child, the womb seemed preternaturally large, and we suspected that it contained another fœtus; but after thorough examination, the uterus was found freed of its contents, but the walls were unnaturally thick.

The child was dead, but no evidence of decomposition. There was considerable ulceration over the cervical portion of the spine. By careful dissection, we found that the bones of the cranium were almost entirely absent, only a small portion of the inferior margins of the occipital, parietal, and frontal bones could be found, and all of the spinous and transverse processes of the vertebra were absent, consequently there was no spinal canal. The back part of the face was covered with a tough membrane, over which their was an imperfect covering of hair. The bones of the face were nearly natural, also those of the extremities. The evidence is almost positive that the child had not been dead but a few hours.

This family are scrofulous; all of the children have sore eyes and ulcers over the body, but not syphilitic. The muscles of the body, except over the upper portion of the spine and head were normal, and sufficiently well developed. The great point of interest in this case, is how did the child live without a nervous centre?

